# Cell–Cell Interactome-Based Pathogenesis and Therapies for Osteosarcoma

**DOI:** 10.3390/cells15060570

**Published:** 2026-03-23

**Authors:** Sriya Neelam, Abdulaziz Hakeem, Yang Yang, Shuying Yang

**Affiliations:** 1 Department of Basic & Translational Sciences, School of Dental Medicine, University of Pennsylvania, 240 South 40th Street, Philadelphia, PA 19104, USA; skneelam@upenn.edu (S.N.); yang0724@upenn.edu (Y.Y.); 2 Department of Basic and Translation Science, School of Dentistry, Umm Al Qura University, Mecca 21955, Saudi Arabia; afhakeem@uqu.edu.sa; 3 The Penn Center for Musculoskeletal Disorders, Perelman School of Medicine, University of Pennsylvania, Philadelphia, PA 19104, USA; 4 Center for Innovation & Precision Dentistry, Penn Dental Medicine and School of Engineering and Applied Sciences, University of Pennsylvania, Philadelphia, PA 19104, USA

**Keywords:** osteosarcoma, cell–cell interactions, tumor microenvironment, metastases, therapy resistance, immune checkpoints, chemotherapy

## Abstract

**Highlights:**

**What are the main findings?**

- The review systematically summarizes how tumor–microenvironment interactions, involving endothelial cells, fibroblasts, stromal cells, immune populations, and osteoclasts, drive osteosarcoma metastasis, therapy resistance, and tumor progression.
- Key molecular and cellular mechanisms, including exosome communication, signaling pathway activation, immune evasion, and vascular remodeling, are identified as central contributors to osteosarcoma aggressiveness.

**What are the implications of the main findings?**

- Understanding these cellular crosstalk mechanisms provides a foundation for developing targeted therapies, immunomodulatory strategies, and microenvironment-focused interventions to overcome therapy resistance and metastatic spread.
- The integrated insights from this review support precision medicine approaches, highlight actionable molecular targets, and guide future research to improve the clinical management of osteosarcoma.

**Abstract:**

Osteosarcoma (OS), the most common primary malignant bone tumor in children and young adults, is characterized by aggressive behavior, frequent metastasis, and resistance to chemotherapy, resulting in poor clinical outcomes. Increasing evidence indicates that OS progression is not solely driven by tumor-intrinsic factors but is strongly influenced by dynamic interactions within the tumor microenvironment (TME). This literature review synthesizes current research on the roles of endothelial cells, fibroblasts, mesenchymal stromal cells, immune populations, and osteoclasts in OS pathogenesis, with emphasis on cell–cell interactions mediated by direct contact, soluble factors, and extracellular vesicles. The studies demonstrate that these interactions promote tumor proliferation, immune evasion, extracellular matrix remodeling, metastatic dissemination, and therapeutic resistance. Adaptive responses of both tumor and stromal cells to environmental stressors contribute to chemoresistance and disease progression. Collectively, our findings highlight the multifactorial nature of OS driven by complex cellular crosstalk within the TME. Understanding these mechanisms highlights the limitations of conventional chemotherapy and encourages the development of combined therapeutic approaches, including targeted therapies, immunomodulation, and microenvironmental interventions. Continued investigation into tumor–microenvironment interactions may facilitate the identification of actionable targets and improve personalized treatment approaches for OS.

## 1. Introduction

Osteosarcoma (OS), the most common primary bone sarcoma, predominantly affects children, adolescents, and young adults, with a smaller secondary incidence peak in older individuals. Current treatment strategies, combining polychemotherapy and surgery, remain suboptimal, as patient survival rates have shown only a minor improvement over recent decades. OS tumors exhibit marked intra- and intertumoral heterogeneity, and the absence of a clearly defined driver mutation has hindered the development of effective targeted therapies. Increasing evidence indicates that OS progression is closely linked to the tumor microenvironment (TME), which comprises bone, stromal, vascular, and immune components. Interactions within this complex ecosystem—including communication mediated by extracellular vesicles (EVs)—promote tumor growth, survival, and dissemination. Elucidating their roles is essential for informing past, current, and emerging therapeutic strategies, including multi-kinase inhibitors and EV-based approaches [1].

Cell–cell interactions within TME influence primary tumor growth, therapeutic resistance, and metastatic dissemination. OS progression is further driven by adaptive responses of tumor and stromal cells to environmental stressors, which contribute to treatment failure and poor prognosis, underscoring the importance of characterizing cellular crosstalk within OS TME [2].

Studies have increasingly focused on dissecting these processes at molecular, cellular, and systemic levels. Analyses of individual cell types’ contribution to OS pathogenesis, chemoresistance, and metastasis may inform the novel therapeutic strategies development.

**Figure 1:**
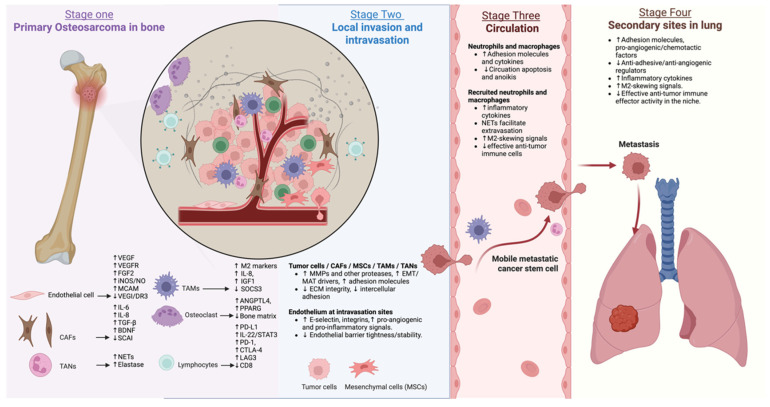
Conceptual framework of osteosarcoma metastasis illustrating stage-specific tumor–TME interactions and associated molecular changes. The diagram traces the progression from primary osteosarcoma in bone (Stage 1), through local invasion and intravasation (Stage 2), circulation of tumor cells in the blood (Stage 3), arrest and extravasation in lung capillaries (Stage 4), to metastatic outgrowth in the lung. At each stage, key tumor-promoting cell types—including endothelial cells, cancer-associated fibroblasts, mesenchymal stromal cells, neutrophils, macrophages, osteoclasts, and lymphocytes—are shown, together with their dominant communication modes (direct contact, soluble factors, extracellular vesicles) and representative molecules that increase (e.g., VEGF-A/VEGF-C, FGF2, IL-6, IL-8, TGF-β, MCAM, IGF-1, NET components, M2-associated cytokines, pro-angiogenic and pro-metastatic noncoding RNAs) or decrease (e.g., VEGI/DR3, SOCS3, invasion-restraining factors, effective cytotoxic T-cell activity) during metastasis. In the primary bone niche, osteosarcoma cells actively promote osteoclast differentiation and activation, driving pathological bone resorption and the release of matrix-stored growth factors that further enhance tumor growth, invasion, and metastatic competence. Also highlighted in this figure is how these coordinated cellular and molecular alterations promote angiogenesis, stemness, extracellular matrix remodeling, immune evasion, bone destruction, chemoresistance, and ultimately lung colonization, thereby prioritizing microenvironmental interactions as potential therapeutic targets in osteosarcoma. Created using BioRender. Yang, S. (2026) https://BioRender.com/9tgb2ng.

Diagnostic-based therapies targeting specific molecular alterations and signaling pathways—together with emerging approaches such as precision medicine, immunotherapy, and metabolic modulation—offer new opportunities [3]. This review synthesizes current evidence on the roles of cell–cell interactions in the TME during OS progression, treatment resistance, and metastatic spread, and evaluates existing and emerging therapeutic strategies to address these challenges (Figure 1).

## 2. Role of Cell–Cell Interactions During OS Progression

### 2.1. Mesenchymal Lineage Cell–OS Crosstalk

#### 2.1.1. Endothelial Cell–OS

Endothelial cells (ECs) actively contribute to OS progression beyond their classical role in blood vessel formation. Through paracrine signaling and EV release, and direct cellular interactions, ECs create supportive niches that enhance tumor growth, stem-like properties, immune modulation, and therapeutic resistance.

Conceptualizing ECs as integral components of the TME reframes angiogenesis as a dynamic, bidirectional process in which tumor cells actively modulate endothelial behavior [4,5,6]. Paracrine and vesicle-mediated signaling are major mechanisms by which ECs influence OS progression. Exosomes derived from human umbilical vein ECs (HUVECs) induce stem-like phenotypes in OS cells by expanding STRO-1 (CD117)-positive populations and upregulating *Oct4* and *Sox2* expression through activation of the Notch pathway. Inhibition of exosome release abrogates these effects, demonstrating a direct EC-tumor signaling axis that promotes intratumoral heterogeneity and aggressive behavior [5].

Angiogenic signaling further reinforces tumor–EC crosstalk. VEGF-C signaling through VEGFR-3 increases inducible nitric oxide synthase (iNOS) activity and nitric oxide production in OS cells, thereby stimulating HUVEC proliferation. This paracrine feedback loop amplifies angiogenesis, supports nutrient delivery, and facilitates tumor dissemination [7]. In parallel, tumor-associated regulators, such as *DEPDC1* and *KIF4A*, enhance VEGF expression and epithelial–mesenchymal transition (EMT) activity in OS cells, promoting endothelial tube formation. Silencing *DEPDC1* activates Hippo signaling and suppresses angiogenesis, illustrating how tumor-derived molecular programs directly shape EC function [8]. Recent single-cell and spatial transcriptomic studies have revealed pronounced endothelial heterogeneity in OS. A distinct population of tip-like ECs has been identified in both primary tumors and metastatic sites. These ECs exhibit angiogenesis- and metastasis-associated gene signatures and elevated MCAM expression. Spatially, they localize adjacent to osteoblast-like tumor cells in metastatic lymph nodes and actively promote endothelial-driven angiogenesis and metastasis [9]. Similarly, endothelial progenitor cells (EPCs) contribute by secreting cytokines. EPC-conditioned media enhances OS cell migration, invasion, and MMP9 expression via PI3K/AKT signaling, while inhibition of VEGF-A or FGF2 attenuates these effects. Consistently, lung metastases from OS patients exhibit increased CD31, VEGF-A, and FGF2 expression, underscoring the role of EPC-mediated angiogenic signaling in metastatic dissemination [10].

Endothelial diversity further influences immune regulation within the TME. Single-cell analyses have identified CLU^+^ endothelial subpopulations at tumor margins that engage malignant cells through integrin-mediated ligand–receptor interactions and promote localized immunosuppression. For example, endothelial Nectin2–TIGIT interactions contribute to CD8^+^ T-cell exhaustion, linking specific endothelial phenotypes to immune evasion [6]. Additional endothelial gene programs, including *COL13A1* and *JAG2*, mediate crosstalk with osteoblastic tumor cells, collectively reinforcing tumor progression [11].

Endothelial–tumor interactions also involve phenotypic plasticity. Under hypoxia, certain OS cells can transdifferentiate into endothelial-like cells, expressing CD31, CD34, and von Willebrand factor and forming tubular networks in vitro and in vivo. This phenomenon supplements conventional angiogenesis and complicates anti-angiogenic therapies by enabling tumor cells to partially substitute for EC function [11]. Epigenetic and noncoding RNA-mediated regulation further modulates endothelial behavior in OS. MicroRNAs and long noncoding RNAs influence angiogenic signaling, including *miR-CT3*-mediated suppression of VEGF-A, *miR-877-3p* targeting of FGF2, and *lncRNA HOXA-AS3*-driven ceRNA interactions (*miR-1286*/*TEAD1*) that affect angiogenesis- and EMT-related pathways [12,13,14]. These layers give insight into endothelial changes in TME and suggest therapeutic targets. OS cells directly interact with ECs, aiding disease progression. Adhesion mechanisms like sLex–E-selectin binding help tumor cell adhesion, aiding migration and invasion. Blocking this interaction reduces OS cell adhesion and invasion in HUVEC models, showing the importance of endothelial adhesion receptors [15].

Endothelial signaling additionally influences therapeutic responsiveness. Endothelial-derived cues upregulate free fatty acid receptors FFA1 and FFA4 in OS cells, enhancing survival under chemotherapeutic stress and linking angiogenic signaling to therapy resistance [11]. Epigenetic modulation of endothelial–tumor signaling pathways can suppress angiogenesis, as demonstrated by restoration of VEGI/DR3 signaling, using hydralazine and sodium valproate [16]. Natural compounds, such as melittin, which inhibit EPC recruitment by blocking SDF-1α/CXCR4 signaling, thereby reducing microvessel density and tumor growth in vivo [17].

ECs act as active regulators of OS pathogenesis rather than passive structural components. By promoting stem-like traits, angiogenesis, immune evasion, vascular invasion, and therapy resistance, ECs play a central role in OS progression and represent promising targets.

#### 2.1.2. Fibroblast–OS

Cancer-associated fibroblasts (CAFs) are key components of the OS TME and profoundly influence tumor progression through direct and indirect cell–cell interactions [18,19,20,21], which are mediated by paracrine signaling, extracellular matrix (ECM) remodeling, and EV-based communication. CAFs secrete cytokines and growth factors, including IL-6, IL-8, and TGF-β, which activate corresponding receptors on OS cells and promote proliferation, survival, and motility [19,20]. This signaling promotes chemoresistance and immune evasion while reinforcing a tumor-supportive microenvironment. Simultaneously, CAF-derived factors influence neighboring stromal cells, including endothelial and immune populations, thereby amplifying tumor-promoting effects within the TME [18].

Direct CAF–OS cell contact further regulates malignant behavior. Cell–cell interactions induce cytoskeletal reorganization and activate signaling pathways that drive epithelial–mesenchymal and mesenchymal–amoeboid transitions (MATs), thereby increasing OS cell migration and invasion [20]. This bidirectional communication establishes a feedback loop in which CAFs respond to tumor-derived cues while actively shaping OS cell phenotypes. CAFs remodel the ECM through deposition and cross-linking of collagen and fibronectin, creating a dense and aligned matrix that provides mechanical support and biochemical guidance for tumor cells [21]. This facilitates directional tumor cell infiltration, thereby contributing to immune suppression and creating a physical barrier that protects the tumor cells from immune attack [22]. Single-cell analyses reveal CAFs as key regulators of the OS environment, showing extensive communication with TME and OS cells. CAF gene signatures categorizes patients into risk groups with differing survival outcomes, immune infiltration patterns, pathway activation states, and drug sensitivities. These findings highlight CAF-driven remodeling’s role in OS progression [22].

Exosome-mediated communication represents an additional mechanism by which CAFs influence OS behavior. CAF-derived small EVs (sEVs) transfer microRNAs, mRNAs, and proteins to OS cells, thereby reprogramming gene expression and enhancing migration, invasion, and survival [21]. For example, exosomal *miR-1228* promotes OS cell motility by suppressing SCAI, a negative regulator of invasion, illustrating how CAFs directly modulate tumor cell behavior through molecular cargo delivery [21]. CAF-derived EVs can also interact with ECs and other stromal cells, indirectly supporting angiogenesis, nutrient delivery, and the establishment of pre-metastatic niches that facilitate distant tumor colonization.

CAFs include inflammatory CAFs (iCAFs) and antigen-presenting CAFs (apCAFs), each contributing uniquely to TME regulation [23]. iCAFs secrete chemokines that regulate leukocyte recruitment and sustain inflammatory signaling, whereas apCAFs interact with immune cells through antigen presentation, potentially modulating immune surveillance and promoting immune tolerance [23]. This heterogeneity underscores the complexity of CAF–tumor interactions and how CAFs promote OS progression, metastasis, and therapy resistance. They also coordinate with immune, endothelial, and mesenchymal stem cells, creating a network that supports tumor survival, vascularization, and spread [21,22]. Through their combined roles in tumor support, EMC remodeling, and immune modulation, CAFs represent promising targets.

#### 2.1.3. Stromal Cell–OS

OS progression is strongly influenced by interactions between tumor cells and the stromal microenvironment. Specifically, mesenchymal stromal cells (MSCs) and CAFs provide a supportive niche for tumor growth. These stromal populations secrete cytokines, chemokines, and EVs that regulate tumor cell proliferation, maintenance of stem-like properties, and motility [24]. By shaping the local microenvironment, MSCs and CAFs also modulate angiogenesis and nerve growth within tumors. For instance, MSC-derived IL-6 and brain-derived neurotrophic factor (BDNF) promote neurite outgrowth and indirectly enhance OS cell proliferation and migration, illustrating how stromal cells influence both tumor cells and the surrounding tissue architecture that favors disease progression [24].

OS-derived stromal cells (OSDCs) display increased sphere-forming capacity and osteogenic differentiation compared with normal MSCs, suggesting that acquisition of cancer stem cell (CSC)-like properties supports tumor-promoting interactions [25]. Importantly, OSDCs frequently lack the complex chromosomal abnormalities characteristic of high-grade OS, indicating that their tumor-supportive effects arise primarily from microenvironmental interactions rather than intrinsic genetic instability [26]. These observations highlight the critical role of the stromal niche in tumor development and progression. MSC heterogeneity is further influenced by anatomical origin within bone tissue. MSCs derived from the femoral diaphysis (FD) and metaphysis (FE) exhibit distinct osteogenic, adipogenic, and chondrogenic differentiation capacities, reflecting intrinsic regional variability [25]. Such differences may influence local TME composition and behavior, thereby affecting OS initiation and progression. Furthermore, environmental stressors, including acidosis or nutrient deprivation, reprogram MSCs toward a protumorigenic state, characterized by increased EV secretion containing microRNAs and proteins that regulate tumor cell metabolism, motility, and survival [26]. This adaptive response underscores the dynamic nature of stromal cell–tumor interactions. In vivo imaging studies also demonstrated that systemically administered human umbilical cord (hUC)-derived MSCs transiently proliferate and distribute across tissues, highlighting the context-dependent behavior and functional plasticity of MSCs [2,25]. These findings indicate that stromal cells are not passive bystanders but active participants whose interactions with tumor cells dynamically shape OS growth, invasion, and potential responses to therapy [2,27].

Collectively, OS progression is also influenced by the surrounding stromal compartment.

By modulating tissue architecture, secreting signaling molecules and EVs, and responding adaptively to environmental stress, MSCs, CAFs, and OSDCs serve as key regulators of tumor cell survival, growth, stemness, and migration. These diverse functions highlight the potential of stromal components as therapeutic targets in OS [24,28].

### 2.2. Immune Cell–OS Crosstalk

#### 2.2.1. Neutrophil–OS

Neutrophils, key regulators of the OS TME, engage in complex interactions with tumor cells, stromal elements, and other immune populations. Tumor-associated neutrophils (TANs) exhibit pronounced phenotypic plasticity, spanning a spectrum ranging from pro-inflammatory, antitumor states to protumorigenic, immunosuppressive phenotypes, depending on local cytokine and chemokine cues [29]. This plasticity enables TANs to influence multiple aspects of tumor biology, including proliferation, angiogenesis, and immune evasion, through both direct cell–cell contact and the secretion of soluble mediators [29].

A major protumorigenic mechanism mediated by TANs involves the formation of neutrophil extracellular traps (NETs), web-like structures composed of DNA, histones, and cytotoxic enzymes that provide a scaffold within the TME, enhancing tumor cell adhesion, migration, and survival [30]. Mechanistically, TAN-derived neutrophil elastase (ELA2) cleaves full-length cyclin E1 into low-molecular-weight cyclin E1 isoforms, promoting OS cell cycle progression and proliferation, thereby directly linking neutrophil activity to tumor growth [31]. Single-cell transcriptomic analyses revealed substantial heterogeneity among TAN populations across primary, recurrent, and metastatic OS lesions. Distinct neutrophil subsets differentially regulate immune cell infiltration, interact with stromal fibroblasts and ECs, and modulate the tumor’s overall immune landscape [32]. Integrative computational analyses incorporating cuproptosis-related biomarkers, including PDHA1 and CDKN2A, alongside immune infiltration profiles, demonstrate that neutrophils are closely associated with tumor metabolic reprogramming and signaling pathways that support cell survival and microenvironment remodeling [33].

Beyond the local effects within the tumor, neutrophils also serve as systemic indicators of tumor-associated inflammation. Circulating neutrophil-based metrics, such as the neutrophil-to-lymphocyte ratio (NLR), reflect systemic immune status and correlate with intratumoral cellular interactions and disease progression [34]. Overall, these findings show that neutrophils are not just passive observers in OS but active modulators of tumor behavior. By controlling proliferation, migration, immune evasion, and metabolic adaptation, TANs help create a microenvironment that promotes tumor growth and metastasis, highlighting their key role in advancing OS aggressiveness.

#### 2.2.2. Macrophage–OS

Tumor-associated macrophages (TAMs) are key regulators of the OS tumor microenvironment (TME), interacting with tumor cells via contact and signaling molecules that influence tumor behavior. M2-like macrophages, a TAM subtype, promote OS progression by secreting cytokines, chemokines, and growth factors that encourage tumor growth, survival, and migration, aiding tumor expansion and metastasis [35]. Specific TAM subpopulations are closely associated with metastatic progression. CD163^+^ EPOR^+^ macrophages are enriched in OS lung metastases and exhibit high expression of M2-associated markers, including CD206 and PD-1. Their close interactions with OS cells reinforce protumorigenic signaling within the metastatic niche.

Cytokine-mediated communication is a key component of this process; for instance, IL-8 produced by TAMs during coculture activates focal adhesion kinase (FAK) signaling in OS cells, thereby increasing proliferation, migration, and invasion [36].

Furthermore, TAMs support osteosarcoma stem cells (OSDCs), a subgroup that initiates tumors and drives tumor heterogeneity and recurrence. Through the *RARRES2*–IGF signaling axis, macrophage-derived insulin-like growth factor 1 (IGF1) sustains OSDC self-renewal and survival, enabling resistance to chemotherapy and promoting tumor relapse [37].

In turn, OS cells secrete HMGB1, which drives macrophage polarization toward the M2 phenotype. These M2 TAMs further enhance tumor cell migration, invasion, and EMT, establishing a reinforcing feedback loop that amplifies tumor aggressiveness [38].

Exosome-mediated communication is a key way TAMs influence OS progression. M2 TAMs release exosomes rich in *miR-221-3p*, suppressing *SOCS3* in OS cells and activating JAK2/STAT3, which promotes proliferation, migration, invasion, and apoptosis resistance [39,40]. In addition, TAM-derived exosomal transfer of PDE4C enhances OS cell proliferation and motility by increasing collagen deposition in the ECM, demonstrating how macrophages remodel the TME to support tumor growth and dissemination [41].

In addition to directly promoting tumor growth, TAMs help tumors evade the immune system by creating an immunosuppressive microenvironment. They secrete inhibitory factors and influence other immune cells, thereby decreasing antitumor immune responses and supporting tumor survival and metastasis [42].

Collectively, TAM–OS interactions are multifaceted and involve soluble mediators, receptor–ligand signaling, exosome-based communication, and immune modulation. These coordinated mechanisms promote OS cell survival, proliferation, stemness, and adaptability, positioning TAMs as key drivers of OS progression, therapy resistance, and metastasis [42].

#### 2.2.3. Lymphocytes

Lymphocytes play a central role in antitumor immunity in OS; however, their activity is strongly influenced by the TME and interactions with other immune and stromal cells. For example, macrophage-mediated efferocytosis increases PD-L1 expression, thereby suppressing CD8^+^ T-cell cytotoxicity, promoting immune tolerance, and limiting effective tumor clearance [43].

*lncRNA AL031775.1* is a T-cell exhaustion-associated molecule that, by upregulating immune checkpoint molecules such as PD-1, CTLA-4, and LAG3, facilitates immune evasion [44]. Advances in immunotherapy aim to counteract these inhibitory mechanisms. Chimeric antigen receptor (CAR)-T cell therapies targeting antigens, such as ALPL-1 or B7-H3, have demonstrated the ability to selectively eliminate OS cells while sparing normal tissues [45]. The efficacy of CAR-T cells can be further enhanced through genetic modifications that improve tumor trafficking (e.g., CXCR5 or CXCR2 expression) or sustain long-term activity and survival, such as IL-7 expression that maintains a stem-like memory phenotype [46,47]. In addition, innate immune cells, including natural killer (NK) cells, further modulate lymphocyte responses by providing early immune cues that influence T-cell recruitment and activation [48]. Ultimately, lymphocyte effectiveness in OS depends on the balance between activating signals and immunosuppressive mechanisms within the TME, which determine whether T cells retain antitumor function or become dysfunctional [49]. For example, in *ABCB1*high/*ABCA1*low osteosarcoma, the TME favors immunosuppression over T-cell activation; elevated *ABCB1* effluxes doxorubicin to confer chemoresistance, while reduced *ABCA1* limits IPP export and subsequent Vγ9Vδ2 T-cell stimulation, collectively rendering intratumoral lymphocytes dysfunctional despite their recruitment [50].

Tumor-derived factors further impair lymphocyte function. IL-22 produced by OS cells activates STAT3 signaling, enhancing tumor proliferation and invasion while concurrently reducing T-cell cytotoxic activity, thereby shifting the immune balance toward tumor progression [51]. Moreover, metabolic interactions within the TME also influence lymphocyte function. *AZIN1*-dependent polyamine synthesis in tumor cells suppresses effector T-cell activity, linking tumor metabolism to immune evasion [52]. Similarly, pleckstrin (PLEK) expression in macrophage-enriched niches coordinates metabolic and immune pathways, shaping microenvironments that can either support or inhibit T-cell activation [53].

In summary, lymphocyte activity in OS is governed by immune checkpoints, cytokine signaling, metabolic constraints, and cellular crosstalk within the TME. Elucidating these regulatory pathways is essential for the rational design of therapies that restore T-cell function and improve antitumor immunity [43].

### 2.3. Osteoclasts

Osteoclasts actively contribute to OS progression through close interaction with tumor cells and the bone microenvironment. Rather than functioning solely as bone-resorbing cells, osteoclasts engage in a dynamic feedback loop with OS cells [54].

Single-cell transcriptomics show heterogeneity in osteoclasts. Some, like highly proliferative C2MKI67^+^ osteoclasts, have stronger interactions with OS cells, greater bone resorption, and support a tumor-permissive microenvironment. These subsets disproportionately promote bone degradation and disease progression [55]. Osteoclast activity is tightly regulated by immune components within the TME. Regulatory CD4^+^ T cells (Tregs) influence osteoclast differentiation and function, and reciprocal interactions between osteoclasts and Tregs shape local immune balance, inflammation, and immune suppression. Through these interactions, osteoclasts contribute to an immune context that can either restrain or promote tumor growth, depending on the prevailing immune signals [56].

Exosome-mediated communication represents an additional regulatory layer in osteoclast–tumor crosstalk. OS-derived exosomes containing *miR-501-3p* promote osteoclast differentiation and activity, enhancing bone resorption and creating conditions favorable for tumor growth. Similarly, noncoding RNA-mediated signaling pathways, such as the *LINC00266-1*/*miR-548c-3p*/*SMAD2* axis, indirectly stimulate osteoclast activity by modifying the local microenvironment while simultaneously supporting OS cell proliferation [57,58]. Other molecular mediators further reinforce osteoclast involvement in OS progression. ANGPTL4 promotes both OS cell proliferation and osteoclastogenesis, demonstrating how tumor-derived factors concurrently enhance tumor growth and bone resorption [59]. In addition, transcriptional regulators, such as PPARG, are activated in osteoclasts during OS progression, highlighting their active role in remodeling the bone microenvironment to support tumor survival and dissemination [56].

Osteoclasts influence the TME through ECM remodeling, release of cytokines and growth factors, and interactions with endothelial and immune cells. These processes increase vascularization, create physical space for tumor expansion, and establish a supportive niche for OS cells [54,55,56,57,58,59]. Beyond direct tumor interactions, osteoclasts can be a predictor of the responsiveness of chemotherapy in young and middle-aged patients. Collectively, osteoclasts contribute to bone degradation and to therapy responsiveness [60].

## 3. Resistance to Treatment

### 3.1. ECs

Tumor-associated ECs contribute significantly to OS resistance to conventional therapies by establishing a pro-survival vascular niche. VEGF-C/VEGFR-3 signaling in MG63 OS cells induces iNOS expression and nitric oxide production, thereby stimulating EC proliferation and reinforcing tumor survival under chemotherapeutic stress [8]. In parallel, endothelial-derived cues reprogram tumor cell metabolism and receptor expression, further enhancing resistance. Specifically, ECs upregulate free fatty acid receptors FFA1 and FFA4 in MG63 cells, increasing resistance to cisplatin-induced cytotoxicity. In contrast, downregulation of these receptors reduces tumor cell viability, underscoring their role in endothelial-driven chemoresistance [61].

Given these mechanisms, therapeutic strategies that simultaneously target tumor and endothelial compartments show promise. Dual-targeted nanomaterials, such as P-Fe_3_O_4_@Pal@HM, induce reactive oxygen species-mediated DNA damage in OS cells while modulating the endothelial microenvironment, thereby bypassing the protective effects of tumor-associated vasculature [4]. Similarly, combination regimens that disrupt endothelial signaling enhance treatment efficacy. For example, ginsenoside Rg3 combined with doxorubicin suppresses both tumor growth and angiogenesis, highlighting the potential of integrated approaches to overcome endothelial-mediated therapy resistance [13].

### 3.2. Fibroblasts

CAFs are central regulators of OS resistance to both chemotherapy and emerging immunotherapies. Neoadjuvant chemotherapy targets malignant cells and remodels the TME, increasing CAF abundance and enhancing their ECM-modifying capacity [21]. These changes promote therapy resistance by supporting tumor cell stemness, restructuring the ECM, and restricting immune cell infiltration, thereby creating a protective niche that allows OS cells to survive cytotoxic stress. Fibroblasts derived from MSCs further exacerbate resistance by secreting cytokines and chemokines, including IL-6, IL-8, and MCP-1, which activate survival signaling pathways and induce MATs in OS cells, increasing invasiveness and drug tolerance [62,63]. In response to tumor-derived signals, CAFs upregulate inflammatory mediators, matrix metalloproteinases, and angiogenic factors, collectively enhancing tumor proliferation, motility, and survival despite chemotherapy or other cytotoxic challenges [21]. This reciprocal interaction reinforces malignant phenotypes and therapeutic resistance.

Single-cell transcriptomics identified a CD36^+^ CAF subset linked to immunosuppression in OS. These CAFs reprogram the TME through the OxLDL–*PPARG*–ANGPTL4 axis, activating *JAK2*–*STAT3* in CD8^+^ T cells and causing T-cell exhaustion, which reduces PD-1 blockade effectiveness. Targeting this pathway with vitamin E reprograms the TME and boosts antitumor responses, showing CAF metabolic crosstalk as a key immune evasion mechanism [64]. CAF-derived exosomes further promote chemoresistance. Exosomal *miR-22-3p* suppresses PTEN and ferroptosis in OS cells, enhancing resistance to cisplatin via the Rab27b/exosomal *miR-22-3p*/*PTEN*/ferroptosis axis. In vivo validation confirms reduced drug sensitivity, identifying exosome-mediated signaling as a critical driver of therapeutic resistance [65].

CAF-mediated resistance also poses a major barrier to immunotherapy. Dense, fibroblast- and ECM-rich stroma physically limits T-cell infiltration, reducing the efficacy of immune-based treatments [66]. For instance, CAF disruption and ECM remodeling are required to enhance T-cell penetration and antitumor activity. Similarly, CAR T-cell therapies face challenges in infiltrating fibroblast-dense tumors, reflecting the combined impact of physical obstruction and CAF-driven immunosuppression [21]. CAF remodels the ECM by depositing and cross-linking collagen and fibronectin, creating aligned tracks that help tumor cell migration, increase metastasis, and block immune infiltration. CAF-derived exosomes with miRNAs, mRNAs, and proteins reprogram tumor and stromal cells, amplifying resistance mechanisms and promoting survival under therapy [66].

CAF heterogeneity further contributes to resistance. Integrative single-cell and bulk RNA sequencing analyses reveal that specific fibroblast subtypes correlate with poor prognosis and reduced chemotherapy responsiveness [63,67].

Some CAF subsets exhibit highly inflammatory profiles that sustain a protumorigenic environment, whereas others directly suppress antitumor immune responses, underscoring their multifaceted roles in OS progression and treatment failure. Collectively, these findings highlight CAFs as active drivers of OS growth, metastasis, and resistance to resistance. By promoting tumor cell plasticity, remodeling ECM architecture, modulating immune activity, and facilitating stromal crosstalk, CAFs establish a protective microenvironment that undermines both conventional and immune-based treatments. Targeting CAF-mediated signaling, ECM remodeling, and immunosuppressive pathways represents a promising strategy to overcome therapy resistance and improve clinical outcomes in OS.

### 3.3. Stromal Cells

Stromal–tumor interactions are major contributors to chemoresistance in OS. MSCs and OSDCs protect tumor cells from environmental stress and chemotherapy EV-mediated signaling and can be reprogrammed or harnessed therapeutically. These cells release EVs that help tumor cells survive better under stress and chemotherapy [26,68]. Stromal signals modulate survival pathways, reduce apoptosis, and help tumor cells adapt. MSC-derived EVs can either promote tumor growth or contain tumor-suppressive microRNAs like *miR-101*, which inhibit OS cell proliferation and migration. This variability underscores the potential of targeting stromal EVs to enhance treatment sensitivity [69,70]. Furthermore, MSCs can act as active carriers for therapy. When loaded with photoactive nanoparticles, MSCs effectively target tumor sites, and localized activation triggers OS cell death without harming MSC viability or migration. In vivo research shows that MSC-mediated delivery notably decreases tumor growth, confirming that stromal cells can be used as targeted therapeutic vectors to counteract microenvironment-driven resistance [27,69].

Drug repurposing strategies illustrate how stromal–tumor dependencies can be therapeutically exploited. A combination of bezafibrate, medroxyprogesterone acetate, and valproic acid (V-BAP) selectively suppresses OS cell survival while sparing normal MSCs by modulating lipid metabolism and reducing fatty acid synthase expression, revealing exploitable metabolic vulnerabilities [28]. Similarly, curcumin-loaded nanoparticles inhibit the protumorigenic effects of MSCs exposed to acidosis, a condition that typically enhances tumor aggressiveness. By suppressing NF-κB-mediated inflammatory signaling, these nanoparticles reduce OS cell stemness, migration, and invasion, indirectly improving chemotherapeutic responsiveness [71].

Beyond modulating stromal signaling, chemoresistant OS subpopulations, including osteosarcoma-initiating (OSi) cells, frequently exhibit CSC-like properties such as self-renewal and enhanced survival, conferring resistance to standard agents, such as doxorubicin [72]. Targeting pathways that maintain this stem-like state can reduce tumorigenicity. For example, inhibition of Rho-associated kinase (ROCK) in OSi cells induces terminal adipogenic differentiation by promoting actin depolymerization and suppressing transcriptional coactivators, thereby reducing stemness and restoring chemosensitivity [72]. These findings demonstrate that altering differentiation status can render resistant tumor cells more vulnerable to therapy.

A comprehensive understanding of stromal–tumor communication and methods to modulate stromal signaling or EV cargo, offers promising strategies to counteract chemoresistance and improve outcomes for OS patients [70].

### 3.4. Neutrophils

Neutrophils are pivotal in modulating OS responses to therapy and contribute to both innate and acquired chemoresistance. Elevated systemic inflammatory markers, including NLR and platelet-to-lymphocyte ratio (PLR), are strongly associated with reduced responsiveness to neoadjuvant chemotherapy [73]. Patients with high pre-treatment NLR frequently exhibit lower rates of pathological complete response (pCR), indicating that neutrophil-mediated inflammation can impair chemotherapeutic efficacy [74]. Similarly, increased PLR correlates with diminished treatment response and poorer overall survival, underscoring the negative prognostic impact of systemic inflammatory status [75]. At the mechanistic level, TANs and NETs establish a protective niche that shields OS cells from both chemotherapeutic agents and cytotoxic immune effector mechanisms [30]. Neutrophil elastase (ELA2), secreted by TANs, promotes the generation of low-molecular-weight cyclin E1 isoform, which accelerates cell cycle progression and confers resistance to therapies targeting proliferating tumor cells [31]. Neutrophil depletion or pharmacological inhibition of ELA2 chemosensitivity highlights neutrophils as active mediators of therapy resistance rather than passive inflammatory markers [31].

Besides modulating OS responses to chemoresistance, preoperative inflammation-based scoring systems further support the clinical relevance of neutrophil activity. Indices, such as the CAR and NLR-based nomograms, independently predict overall survival and treatment outcomes in OS [76]. In addition, the Naples Prognostic Score (NPS), which integrates neutrophil counts with other systemic parameters, enables multidimensional risk stratification and identifies patients more likely to exhibit resistance to standard chemotherapy regimens [77].

Collectively, these findings indicate that neutrophils and systemic inflammation are not merely linked to poor prognosis but also play an active role in therapy resistance in OS.

### 3.5. Macrophages

TAMs are major contributors to OS resistance to chemotherapy, radiotherapy, and immunotherapy. M2-like TAMs establish an immunosuppressive TME that protects malignant cells from cytotoxic drugs and immune-mediated attacks [35]. NF-κB signaling in OS cells promotes TAM recruitment and sustains their polarization toward the M2 phenotype, creating a self-reinforcing feedback loop that enhances tumor survival and reduces therapeutic efficacy [78].

TAMs also exert direct pro-survival effects on OS cells. IL-8 released by TAMs activates FAK signaling in tumor cells, promoting proliferation, migration, and survival under treatment stress [38]. In parallel, TAM-derived insulin-like growth factor 1 (IGF1) supports OS stem cells through the *RARRES2* axis. These stem-like tumor cells exhibit enhanced therapy resistance and contribute to disease recurrence after treatment [38]. TAMs additionally influence immunotherapy efficacy. Elevated PDE4C expression within tumors correlates with reduced PD-L1 expression and impaired immune activation, potentially limiting the response to immune checkpoint blockade [41]. Computational and transcriptomic analyses have identified TAM-associated genes, such as *BNIP3*, that simultaneously regulate tumor growth and immune suppression, highlighting potential targets for precision-based therapeutic interventions [79].

Targeting TAM activity has therefore emerged as a promising therapeutic strategy. Combination approaches that TAM polarization alongside chemotherapy have demonstrated improved antitumor effects. For example, curcumol combined with cisplatin inhibits M2 polarization, sensitizes tumor cells to chemotherapy, and suppresses tumor growth [80]. Similarly, targeting the macrophage receptor MARCO reprograms TAMs toward a pro-inflammatory M1 phenotype, increasing antitumor cytokine release and enhancing chemosensitivity [80]. Moreover, exosome-mediated signaling represents another critical mechanism of TAM-driven therapy resistance. M2-polarized TAMs release exosomes enriched in *miR-221-3p*, which suppresses *SOCS3* expression in OS cells and activates the *JAK2*/*STAT3* pathway, thereby promoting tumor cell survival, proliferation, and migration during treatment [40]. TAM-derived exosomal PDE4C further enhances OS cell proliferation and motility by remodeling the ECM, facilitating therapeutic escape [41].

Collectively, TAMs function as central regulators of therapy resistance in OS by sustaining immunosuppression, supporting stem-like tumor cell populations, modulating immune responses, and delivering resistance-promoting exosomal cargo. These multifaceted roles position TAMs as critical therapeutic targets for strategies to overcome treatment resistance and improve clinical outcomes in OS [80,81].

### 3.6. Lymphocytes

Lymphocyte-mediated antitumor responses in OS are compromised by multiple resistance mechanisms that limit the efficacy of conventional chemotherapy and immunotherapy. A key contributor is the dysregulation of drug transporters in tumor cells. High *ABCB1* expression promotes doxorubicin efflux, reducing intracellular drug accumulation and limiting immunogenic cell death (ICD), a process essential for effective activation of cytotoxic lymphocytes. Concurrently, reduced *ABCA1* expression impairs the recruitment and activation of Vγ9Vδ2 T cells, a highly cytotoxic innate-like T-cell subset, thereby promoting chemo-immune resistance [50]. In addition, OS-derived granulocyte colony-stimulating factor (G-CSF) drives the expansion of myeloid-derived suppressor cells (MDSCs), which inhibit CAR-T cell function, impair antigen presentation, and suppress T-cell activation within the TME [82].

T-cell exhaustion are a major barrier to effective immunotherapy in OS. This dysfunctional state is characterized by reduced cytokine production, impaired cytotoxicity, and sustained expression of immune checkpoint molecules. Exhaustion is further reinforced by high expression of *HHLA2*, a B7-family immune checkpoint that is frequently upregulated in OS, particularly in metastatic sites. Elevated *HHLA2* levels are associated with reduced T-cell infiltration, enhanced immune evasion, and poorer clinical outcomes, underscoring its relevance as a therapeutic obstacle [83].

Tumor-intrinsic signaling pathways further enhance immune resistance. Cytokines, such as IL-22, activate *STAT3* signaling, promoting OS cell survival, proliferation, and invasion while concurrently suppressing T cell expansion and effector function [51]. In parallel, metabolic reprogramming in OS cells, including *AZIN1*-dependent polyamine synthesis and activation of Ras/AKT/mTOR and ERK/HIF-1α pathways, supports tumor growth but deprives effector lymphocytes of metabolic resources required for effective cytotoxic responses [50,52]. Overexpression of T-LAK leukemia-associated antigen kinase (TOPK) represents an additional resistance mechanism that enhances proliferation, metastasis, and chemoresistance; pharmacological inhibition of TOPK restores lymphocyte sensitivity and improves responses to cytotoxic therapies [84].

Collectively, these findings indicate that lymphocyte-associated resistance in OS arises from the convergence of tumor-intrinsic defenses, metabolic constraints, immune checkpoint activation, and suppressive stromal constraints. This complexity highlights the need for integrated therapeutic strategies that simultaneously target tumor cells and the immunosuppressive microenvironment to restore effective antitumor immunity [85,86].

### 3.7. Osteoclasts

Osteoclasts are key players in bone remodeling in OS and are closely involved in tumor–bone interactions. Evidence indicates that factors released by OS cells can directly affect both osteoblast differentiation and osteoclastogenesis. For instance, Semaphorin 3A (Sema3A) promotes osteoblastic differentiation in osteosarcoma cell lines while reducing cell migration and osteoclast formation in vitro. In vivo, exogenous Sema3A increased bone volume in tumor-bearing models, whereas tumor-derived Sema3A impacted ectopic bone formation via DKK1/β-catenin signaling. These results emphasize the complex, bidirectional regulation between bone formation and resorption in OS and illustrate how controlling osteoclast activity is closely linked to tumor development and bone remodeling processes [87].

OS cells actively modulate signaling pathways that promote tumor survival while simultaneously enhancing osteoclast differentiation. Activation of the NF-κB pathway in tumor cells supports resistance to chemotherapy and stimulates osteoclast activity, reinforcing a microenvironment that favors tumor persistence under therapeutic pressure [56]. Moreover, noncoding RNAs in osteoclast-associated chemoresistance. The long noncoding RNA *LINC00266-1* regulated the *miR-548c-3p*/*SMAD2* axis, promoting OS cell survival, proliferation, and metastatic potential. By modifying TME, this pathway indirectly enhances osteoclast activity and contributes to tumor resistance to chemotherapy [58].

Several therapeutic agents targeting osteoclast activity have demonstrated beneficial effects on chemotherapy efficacy. Bovine lactoferrin suppresses the expression of pro-osteoclastogenic and inflammatory mediators, including IL-1β, IL-6, and RANKL, and inhibits NF-κB signaling in OS cells. This dual action reduces osteoclast activation and enhances the antitumor effects of chemotherapeutic agents [88]. Similarly, RANKL-targeting therapies, such as denosumab, inhibit osteoclast-mediated bone resorption and can indirectly improve chemotherapy outcomes by limiting bone destruction and tumor-supportive signaling [58,89].

Beyond therapies targeting RANKL, bisphosphonates have also been investigated as osteoclast-focused treatments in osteosarcoma. Zoledronic acid (ZOL), a widely used inhibitor of bone resorption, has demonstrated additional benefits when combined with other treatments. For instance, combining ZOL with the tumor-specific oncolytic adenovirus OBP-301 increased apoptosis in osteosarcoma cells by suppressing the anti-apoptotic protein MCL1, while also decreasing tumor-induced osteoclast activation.

In orthotopic models, this combination was more effective at limiting tumor growth and bone destruction than either treatment alone. These findings support the idea that concurrently targeting osteoclast activity and tumor survival pathways could enhance therapeutic outcomes and help manage disease progression OS [90,91].

Overall, osteoclasts contribute to chemotherapy resistance in OS via bone remodeling, signaling, and interactions with tumor cells. Targeting osteoclasts or disrupting osteoclast–tumor signaling—using RANKL inhibitors, anti-inflammatory agents, and noncoding RNA—offers promising strategies to improve chemotherapy response and reduce treatment failure [90,91].

## 4. Metastatic Dissemination of OS

### 4.1. ECs

Metastatic dissemination of OS is strongly dependent on interactions between tumor cells and ECs, which regulate vascular permeability, angiogenesis, and tumor cell entry into the circulation. ECs provide both structural scaffolding and biochemical signals that facilitate intravasation, survival in the bloodstream, and colonization of distant organs, positioning them as central regulators of metastatic efficiency [4,5,6,7].

Endothelial contributions to OS metastasis are highly heterogeneous. Single-cell RNA sequencing has identified a CLU^+^ endothelial subpopulation enriched at invasive tumor margins that promotes local tumor progression and immune evasion [6]. These ECs express ligands such as NECTIN2 and ICAMs that interact with tumor and immune cells to induce T-cell exhaustion and weaken immune surveillance, indirectly enhancing metastatic potential. Their preferential localization near vascular interfaces suggests a role in facilitating both tumor cell dissemination and metastatic seeding [6]. Beyond structural remodeling, EC-derived signals directly enhance tumor aggressiveness. Exosomes released by HUVECs activate Notch signaling in OS cells, upregulating stemness-associated markers such as *OCT4* and *SOX2* and increasing migratory capacity [5]. These interactions promote an EMT-like phenotype that primes tumor cells for vascular invasion. The VEGF-C/VEGFR-3/iNOS signaling axis establishes a paracrine feedback loop in which tumor-derived VEGF-C stimulates endothelial nitric oxide production, promotes vessel expansion, and increases intratumoral entry points for circulating tumor cells [8]. In addition to the resident ECs, circulating EPCs contribute to metastatic niche formation. Inhibition of EPC using melittin suppresses SDF-1α/CXCR4 signaling, reduces microvessel density, and decreases metastatic burden [17], highlighting this pathway as a potential target for limiting secondary tumor colonization.

Endothelial–tumor crosstalk is further modulated by metabolic and molecular regulators. Combined treatment with ginsenoside Rg3 and doxorubicin suppresses the mTOR/HIF-1α/VEGF pathway, reducing angiogenesis and microvessel density and limiting metastatic dissemination [92]. Conversely, loss of anti-angiogenic microRNAs, such as *miR-CT3*, promotes endothelial activation and microvascular remodeling, thereby facilitating tumor invasion and spread [12]. Moreover, tumor plasticity adds another layer of complexity to endothelial-mediated metastasis. Under hypoxic conditions, OS cells, such as MNNG/HOS, acquire endothelial-like features, express CD31, CD34, and vWF, and form tubular networks in vitro and in vivo [11]. This phenomenon, known as vascular mimicry, enables tumor cells to maintain perfusion independently of canonical angiogenesis, complicating anti-angiogenic therapeutic strategies [15]. Single-cell profiling of OS lung metastases further underscores the complexity of endothelial-associated metastatic niches.

These lesions contain heterogeneous immune populations, including CD8^+^ T cells with relatively low checkpoint expression, as well as malignant subclusters marked by CD63, suggesting context-dependent therapeutic vulnerabilities [11].

Collectively, these findings demonstrate that ECs and endothelial-like intermediates actively coordinate OS metastasis by establishing vascular routes, modulating immune responses, and enhancing tumor cell adaptability. Targeting endothelial-driven processes, such as VEGF signaling, exosomal Notch activation, vascular mimicry, or SDF-1α/CXCR4-mediated recruitment, represents a promising strategy to limit both local invasion and distant metastatic spread.

### 4.2. Fibroblasts

CAFs play a central role in promoting OS metastasis by directly modulating tumor cell behavior and remodeling the surrounding stroma. CAFs communicate with OS cells primarily through paracrine signaling, releasing cytokines and chemokines such as IL-6, IL-8, and MCP-1 that induce MATs, thereby enhancing tumor cell motility, invasiveness, and transendothelial migration [63,93]. These interactions increase the migratory capacity of OS cells, support survival during circulation, and facilitate colonization of the distant organs, particularly the lungs [21].

Recent studies have identified fibroblast growth factor 23 (FGF-23) as a key mediator of CAF-driven metastasis. FGF-23 promotes OS cell migration by upregulating lysyl oxidase-like 2 (LOXL2) through ERK-, p38-, and JNK-dependent signaling while concurrently suppressing *miR-4463* expression. Functional validation demonstrates that LOXL2 knockdown markedly reduces OS cell migration, highlighting the FGF-23/LOXL2 axis as a critical pathway linking CAF-associated signaling to metastatic dissemination [94]. CAF-mediated regulation of tumor cell-intrinsic signaling also contributes to metastasis. The super-enhancer-associated long noncoding RNA *ZMIZ1-AS1* promotes OS progression and lung metastasis through ALKBH5-mediated m^6^A demethylation, thereby stabilizing *ZMIZ1-AS1* and facilitating its interaction with PTBP1. This interaction enhances FGFR1 mRNA stability and nuclear translocation, driving tumor cell proliferation, migration, and invasion. Combined inhibition of ALKBH5 and FGFR1 effectively suppresses this metastatic axis, demonstrating how CAF-associated regulatory networks indirectly amplify tumor aggressiveness [95].

Single-cell analyses additionally reveal that CAF heterogeneity influences metastatic behavior. Distinct fibroblast subtypes, including iCAFs and apCAFs, facilitate immune evasion and ECM remodeling, thereby creating permissive niches for tumor intravasation and extravasation [23,67]. By altering stromal composition and adhesion dynamics, these CAF subsets support vascular invasion and metastatic seeding [67]. Exosome-mediated communication represents an additional mechanism by which CAFs facilitate metastatic spread. CAF-derived exosomes containing miRNAs, including miR-1228, suppress tumor suppressor pathways, promote cytoskeletal remodeling, and enhance OS cell migration and invasion [96]. Through vesicle-mediated signaling, CAFs remodel the TME and contribute to the establishment of pre-metastatic niches at secondary sites, most notably in the lungs [21]. In addition to CAFs, fibroblasts derived from SCs enhance OS metastatic competence by secreting growth factors and activating pro-survival signaling pathways, such as PI3K/mTOR/S6K, which promote cytoskeletal reorganization and EMT-like phenotypes [21].

These findings show that CAF-driven cytokine signaling, exosome communication, and ECM remodeling work together to coordinate various stages of OS metastatic spread. The diverse functionality and adaptability of CAFs highlight their crucial role in regulating metastasis and suggest that strategies targeting fibroblasts could be effective in limiting OS progression.

### 4.3. Stromal Cells

Metastatic dissemination of OS, particularly to the lungs, is strongly influenced by interactions between tumor cells and the surrounding stromal microenvironment. MSCs and CAFs contribute to this process by secreting cytokines, including IL-6 and TGFβ1, that enhance OS cell stemness, migration, and metastatic potential [24]. In addition to soluble mediators, MSC-derived EVs transport microRNAs, proteins, and signaling molecules that regulate invasion, survival in circulation, and colonization of distant organs, establishing a direct mechanistic link between stromal support and metastatic progression [26,69].

Prometastatic signaling is further amplified by environmental stressors within the TME, such as hypoxia and extracellular acidosis. Under these conditions, OS cells exhibit increased motility, enhanced invasive capacity, and greater potential for distant colonization [71,97]. Notably, preclinical studies using EVs enriched with tumor-suppressive microRNAs, such as miR-101, demonstrate significant inhibition of lung metastasis, highlighting stromal EVs as both drivers of metastasis and potential therapeutic tools [69]. In addition to EVs, emerging biomaterial-based strategies have been developed to limit metastasis while supporting tissue repair.

Magnetic mesoporous calcium silicate/chitosan scaffolds enable localized bone regeneration and deliver antitumor therapy through controlled drug release and photothermal ablation. These multifunctional platforms reduce residual tumor burden and may limit the contribution of surviving OS cells to metastatic dissemination [27]. Moreover, non-invasive imaging studies further indicate that the biodistribution and functional impact of MSCs and their EVs are influenced by the route of administration and host immune status. These findings suggest that both local tumor control and systemic metastasis prevention can be modulated by optimizing stromal cell- or vesicle-based delivery strategies, providing important guidance for therapeutic design [27,69].

Importantly, OS-associated stromal cells typically lack the complex genetic alterations seen in high-grade tumor cells, yet retain the capacity to facilitate metastatic colonization [98], underscoring that functional stromal support, rather than tumor-intrinsic mutations alone, plays a critical role in OS dissemination. Collectively, these findings emphasize the need for therapeutic strategies that target both tumor cells and their supportive stromal microenvironments to effectively prevent or limit OS metastasis.

### 4.4. Neutrophils

Neutrophils actively promote OS metastasis through coordinated local TME remodeling and systemic inflammatory mechanisms. TANs are enriched in metastatic and recurrent OS lesions, where their presence correlates with reduced stromal and immune scores and increased tumor purity, collectively creating conditions that favor tumor cell dissemination [29]. Clinically, elevated neutrophil counts and increased PLR prior to treatment have been identified as independent predictors of metastatic progression, underscoring the contribution of systemic neutrophil-mediated inflammation to OS metastasis [99].

A key mechanistic pathway involves NETs, which facilitate metastasis by physically capturing circulating tumor cells (CTCs), enhancing their adhesion to endothelial surfaces, and promoting extravasation into secondary organs, such as the lungs [100]. Evidence from sarcoma studies, including Ewing’s sarcoma, demonstrates that NET formation is associated with metastatic disease, poor chemotherapy response, and reduced survival, suggesting that NET-mediated dissemination is a conserved mechanism across sarcoma subtypes [30]. These findings indicate that NETs function as structural scaffolds and as active regulators of tumor cell survival and colonization at distant sites.

At the molecular level, neutrophil-derived enzymes and signaling molecules, such as ELA2, activate pro-metastatic pathways in OS cells, including PI3K-AKT and HIF-1 signaling. Activation of these pathways enhances tumor cell invasion, migration, and colonization of distant tissues [31]. Through these combined local and systemic effects, neutrophils function as both facilitators and amplifiers of metastatic dissemination [31].

Prognostic models incorporating neutrophil-related parameters further support their role in metastasis. Pre-treatment indices based on neutrophil count have shown strong predictive value for overall survival and lung metastasis-free survival [101]. Additional systemic inflammation markers, including NLR, PLR, neutrophil-to-platelet score (NPS), and systemic inflammation index (SII), provide complementary metrics for stratifying metastatic risk and informing individualized therapeutic strategies [75,102].

Collectively, these data indicate that neutrophils are central regulators of OS metastasis. Their involvement in TME remodeling, NET formation, and systemic inflammatory signaling highlights neutrophil-associated pathways as potential therapeutic targets for limiting metastatic spread and improving clinical outcomes.

### 4.5. Macrophages

TAMs are central regulators of OS metastasis, particularly to the lungs, the most common site of dissemination, and a major determinant of poor clinical outcomes. Macrophage polarization critically influences metastatic potential. Studies have shown that M2-like TAMs preferentially accumulate in metastatic lesions, where they support OS cell survival, migration, and invasion [35,81]. In experimental models, metastatic OS cells secrete CCL2, which recruits M2 macrophages to the lungs and establishes a pro-metastatic niche that facilitates tumor colonization [81]. Moreover, HMGB1 promotes M2-like polarization of TAMs, which, in turn, enhances OS cell migration, invasion, and EMT, thereby forming a reinforcing feedback loop that favors metastatic spread. At metastatic sites, TAMs further facilitate immune evasion. In the lung microenvironment, M2-like macrophages cooperate with myeloid-derived suppressor cells to create an immune-excluded niches that limit T cell infiltration, allowing disseminated tumor cells to survive and expand [103].

Exosome-mediated communication represents an additional mechanism through which TAMs promote metastasis. Exosomes released by M2-polarized TAMs contain *miR-221-3p*, which activates the JAK2/STAT3 pathway in OS cells, thereby increasing motility and invasiveness [40]. Similarly, PDE4C delivered via TAM-derived exosomes enhances tumor cell migration and remodels the ECM, creating a microenvironment conducive to metastatic progression [41].

Not all macrophage populations support tumor spread. Some subsets, such as C1Q+ macrophages, are associated with antitumor immunity and improved survival. Patrolling monocytes reduce lung metastasis, showing myeloid cell diversity in OS [104,105]. Tumor-intrinsic factors also influence macrophage recruitment; hyperactivation of MYC suppresses CSF1 expression, reducing macrophage infiltration and altering the metastatic landscape [39].

Overall, TAMs facilitate OS metastasis by recruiting cells via chemokines, promoting pro-tumor phenotypes, signaling through exosomes, and inhibiting local antitumor immune responses. These diverse functions position TAMs as key drivers of OS spread, emphasizing macrophage-targeted therapies as a promising strategy to prevent OS progression and enhance patient outcomes [40].

### 4.6. Lymphocytes

Metastatic dissemination of OS is closely linked to lymphocyte dysfunction and multilayered immune evasion mechanisms that enable tumor cells to survive circulation and secondary lesions. Systemic inflammation is a major determinant of metastatic behavior. Clinical indicators, including elevated NLR, C-reactive protein (CRP), and poor Glasgow prognostic scores, are strongly associated with reduced overall survival, reflecting an imbalance between inflammatory signaling and effective lymphocyte-mediated immunity that favors tumor dissemination [34,106].

At the tumor level, OS-derived IL-22 activates STAT3 signaling, enhancing tumor cell motility and invasiveness, while concurrently suppressing lymphocyte-mediated immune surveillance, thereby promoting metastatic potential [51]. Additionally, metabolic reprogramming within the TME further undermines lymphocyte function during metastasis. Tumor cell clusters with elevated PLEK expression exhibit increased glycolytic and oxidative phosphorylation activity, creating energy-rich niches that suppress lymphocyte effector function while supporting tumor survival and dissemination [53]. In addition, AZIN1-driven polyamine synthesis impairs effector T cell activity at metastatic sites, reducing immune pressure and facilitating the establishment of secondary tumors [52].

Meanwhile, immune checkpoint dysregulation contributes to metastatic immune escape. The B7-family checkpoint molecule HHLA2 is expressed at higher levels in metastatic lesions than in primary tumors, where it induces profound local immunosuppression and restricts T cell infiltration, facilitating evasion of cytotoxic immune responses [83]. To counteract these barriers, engineered T-cell therapies have incorporated chemokine receptors, such as CXCR2 and CXCR5, which improve CAR-T cell trafficking to metastatic sites and enhance local antitumor activity [46,47]. In parallel, NK cells contribute to early immune control of disseminated tumor cells, particularly when their activity is augmented through adoptive transfer or antibody-dependent mechanisms [83].

Together, these findings demonstrate that OS metastasis arises from the convergence of systemic inflammation, immune checkpoint-mediated suppression, metabolic adaptation, and lymphocyte dysfunction. Rather than a single immune defect, the integration of these processes determines whether disseminated tumor cells are eliminated or successfully colonize distant tissues [43].

### 4.7. Osteoclasts

Osteoclast-driven bone resorption contributes to local OS growth and to metastatic dissemination. By degrading mineralized bone, osteoclasts release matrix-bound growth factors and cytokines and stimulate surrounding osteoblastic and stromal cells, which can increase RANKL expression. Simultaneously, this resorption creates space that allows tumor cell invasion into adjacent tissues and enter the circulation, thereby facilitating metastasis [54]. Additionally, molecules like ANGPTL4 enhance both osteoclast differentiation and OS cell migration, directly connecting bone remodeling processes to metastatic potential [56,57].

Osteoclasts also shape the metastatic microenvironment through interactions with immune pathways. Under inflammatory conditions, osteoclasts secrete IL-27, which modulates local immune responses and may contribute to the establishment of a niche permissive for tumor colonization [107]. In parallel, OS cells promote bone degradation through osteoclast-independent mechanisms, including expression of uPARAP/Endo180, indicating that tumor-intrinsic and osteoclast-mediated pathways cooperate to enhance invasion and metastatic risk [108].

Regulatory feedback loops further reinforce osteoclast–tumor interactions. The *LINC00266-1*/*miR-548c-3p*/*SMAD2* signaling axis enhances OS cell proliferation while simultaneously modifying the tumor microenvironment to promote osteoclast-mediated bone degradation. This coordinated regulation facilitates tumor escape from the primary site and supports metastatic seeding at distant locations [58].

These findings identify osteoclasts as main drivers of OS metastasis, promoting invasion, dissemination, and colonization via bone resorption, growth factor release, immune modulation, and cooperation with tumor pathways. Targeting osteoclasts or their regulation offers promising strategies to limit metastasis [109,110]. Currently, targeting osteoclast activity has demonstrated efficacy in limiting metastatic progression in preclinical models. The bisphosphonate zoledronic acid suppresses osteoclast activation, reduces bone destruction, and decreases metastatic burden in OS [91]. Similarly, Sema3A inhibits osteoclastogenesis and abnormal bone remodeling, thereby limiting the formation of a microenvironment that supports tumor dissemination [56].

## 5. Integrated Molecular Signaling Pathways and Non-Coding RNA Regulation of the Osteosarcoma Tumor Microenvironment

While the genetic makeup of OS has been well studied, with frequent losses of *TP53* and *RB1*, as well as alterations in Wnt, NF-KB, and PI3K/AKT pathways, TME plays a crucial role in the disease’s progression, metastasis, and resistance to therapy. The OS TME is comprised of various cell types including osteoblasts, osteoclasts, MSCs, immune cells, endothelial cells, and a mineralized ECM. These elements offer structural and biochemical signals that influence oncogenic pathways in OS cells and create a feedback system that remodels the microenvironment to promote tumor growth, blood vessel formation, immune evasion, and spread. The TME is marked by high metabolic activity, rich blood supply, and hypoxic regions, all of which enhance tumor invasion and metastasis potential [111,112].

TME-derived factors regulate several key oncogenic pathways in OS cells, including PI3K/AKT/mTOR, MAPK, Wnt/β-catenin, NFκB, and TGFβ/BMP. MSC-derived chemokines such as CXCL12/SDF1 and cytokines including IL6 and VEGF engage CXCR4 and STAT3/NFκB signaling in OS cells, promoting migration, invasion, and survival. Conversely, OS cells secrete RANKL to stimulate osteoclast-mediated bone resorption and release factors that polarize TAMs toward immunosuppressive M2 phenotypes, recruit MDSCs, and modulate T cell infiltration, creating an immune-privileged niche. The combined effect of these interactions forms a vicious cycle where TME components drive oncogenic signaling and are reprogrammed to sustain tumor growth, metastasis, and chemoresistance. This remodels TME through angiogenesis, immunosuppression, and osteolysis [111,112,113].

Noncoding RNAs (ncRNAs) are central regulators of OS pathogenesis and mediators of TME crosstalk. Small noncoding RNAs (sncRNAs), including microRNAs (miRNAs), piRNAs, and small nucleolar RNAs (snoRNAs), are differentially expressed in OS cells compared with mesenchymal stem cells, influencing proliferation, migration, and invasion. For instance, overexpression of specific piRNAs, snoRNAs, snRNAs, and miRNAs (hsa-miR-369-5p) inhibits OS cell proliferation and migration, highlighting their functional roles in tumor biology [114]. miRNAs can act as OncomiRs or tumor suppressors by regulating key oncogenic pathways, including PI3K/AKT, Wnt/β-catenin, and NFκB. Similarly, long noncoding RNAs (lncRNAs), such as *MALAT1*, *HOTAIR*, *TUG1*, and *CASC2*, function as competing endogenous RNAs, scaffolds for chromatin modifiers, and transcriptional regulators, controlling proliferation, epithelial–mesenchymal transition (EMT), metastasis, and drug resistance [115]. These lncRNAs often correlate with TME-related gene signatures, immune infiltration, and stromal scores, demonstrating their dual role in intrinsic tumor signaling and modulation of the surrounding microenvironment [113]. Circular RNAs (circRNAs) contribute to this network by sponging miRNAs, modulating OS signaling pathways, and being packaged into extracellular vesicles (EVs) to influence distant premetastatic niches.

EV-mediated transfer of ncRNAs is vital for OS–TME communication. OS-derived EVs carry miRNAs and lncRNAs that reprogram MSCs, endothelial cells, and immune cells, promoting angiogenesis, immune evasion, and metastatic niche formation. For instance, ncRNAs transferred from OS cells can directly influence TAM polarization toward a pro-tumorigenic phenotype and promote MDSC expansion. Meanwhile, EVs from MSCs or immune cells deliver ncRNAs that regulate OS cell invasion, stemness, and chemoresistance. This underscores ncRNAs as both intracellular regulators of oncogenic signaling and intercellular messengers orchestrating TME remodeling [111,112,113].

TME-associated ncRNAs are biomarkers and targets in OS. High levels of specific TME-related lncRNAs are associated with poor prognosis and distinct immune infiltration, reflecting an immunosuppressive and pro-metastatic microenvironment. Targeting these ncRNAs, alone or with immunotherapy, could disrupt tumor-TME interactions and improve treatment in metastatic or recurrent OS [112,113]. Single-cell sequencing, spatial transcriptomics, and multi-omics integration are expected to enhance our understanding of ncRNA–TME interactions and facilitate the development of personalized treatment strategies that simultaneously target OS cells and their supportive microenvironment [111].

Therefore, the molecular signaling pathways involved in osteosarcoma are closely linked to their environment within the TME, with noncoding RNAs acting as key regulators and communicators in this dynamic interaction. By combining information on genetic changes, oncogenic signals, and ncRNA-mediated exchanges, OS cells leverage the bone and immune microenvironments to promote growth, invasion, metastasis, and resistance to therapy. Understanding these interconnected networks is essential for developing advanced TME-targeted treatments and ncRNA-based therapies that could enhance patient outcomes for osteosarcoma.

## 6. Therapeutic Approaches to Osteosarcoma

### 6.1. Diagnostic-Based Precision Therapies

OS is characterized by marked genetic instability. In contrast to sarcomas defined by recurrent chromosomal translocations, OS exhibits extensive DNA copy-number variations, chromosomal gains and losses, and frequent alterations in key tumor suppressor genes, including *TP53*, *RB1*, and *ATRX*, along with activation of oncogenes such as MYC and MDM2 [116]. This molecular heterogeneity underlies OS pathogenesis and provides the rationale for diagnostic-based precision therapy, in which treatment strategies are tailored to the molecular profile of an individual tumor.

Conventional diagnostic methods, like imaging and histopathology, are crucial for tumor detection, staging, and surgery. However, advances in molecular diagnostics—such as next-generation sequencing, liquid biopsies, and gene panels—allow for identifying actionable genomic changes and tracking tumor evolution [116].

These technologies can detect dysregulation in pathways, thereby informing the selection of targeted and immunomodulatory therapies. Collectively, diagnostic-based approaches support a shift from uniform treatment paradigms toward precision medicine strategies that optimize therapeutic efficacy while minimizing unnecessary toxicity.

### 6.2. Mechanism-Based Therapies

**Figure 2:**
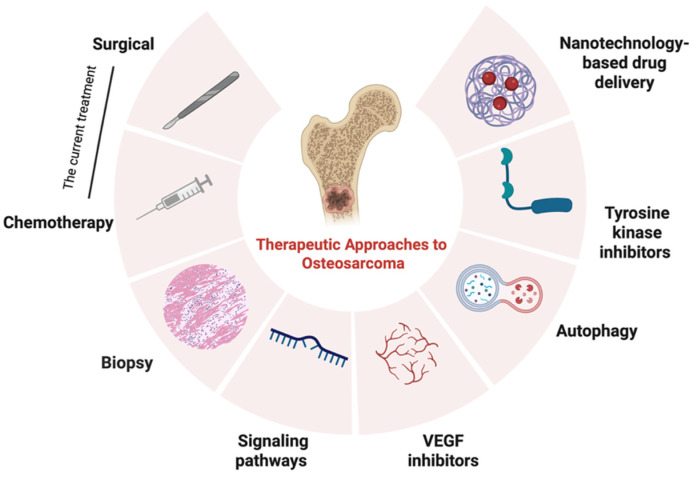
Overview of the current and emerging therapeutic strategies in osteosarcoma. The schematic integrates traditional diagnostics and treatment with modern precision-medicine approaches, highlighting how next-generation sequencing, liquid biopsies, and targeted gene panels identify actionable alterations (for example, in PI3K/AKT/mTOR, HER2, and immune checkpoints) to guide individualized therapy selection. Current standard care—limb-sparing surgery plus multi-agent chemotherapy (doxorubicin, cisplatin, methotrexate)—is shown alongside key signaling pathways (PI3K/AKT/mTOR, JAK/STAT, Wnt/β-catenin, NOTCH, Hedgehog, NF-κB) and targeted agents under investigation, including TKIs (e.g., DCC-2036), MCL-1/BCL-2 family inhibitors, and VEGF-directed anti-angiogenic therapies. The central influence of the tumor microenvironment is depicted through stromal fibroblasts, immunosuppressive macrophages, and inhibitory T cells (notably TIGIT^+^ T cells), as well as autophagy-dependent survival mechanisms that modulate response to chemotherapy and targeted drugs. Future directions emphasize combination strategies that pair pathway inhibitors, dual VEGF/mTOR blockade, immunotherapies (checkpoint inhibitors, CAR-T cells, macrophage reprogramming, TIGIT targeting), nanotechnology-based drug delivery, autophagy modulation, and metabolic targeting to overcome heterogeneity, limit resistance, and reduce toxicity in osteosarcoma. Created in BioRender. Yang, S. (2026) https://BioRender.com/g7glfxb.

Current OS treatment consists of surgical resection combined with systemic chemotherapy, most commonly doxorubicin, cisplatin, and methotrexate (Figure 2). While this approach is effective for localized disease, outcomes remain poor in patients with metastatic or recurrent OS, highlighting the need for more precise, molecularly guided therapeutic strategies [116].

Multiple signaling pathways drive OS growth, progression, and therapy resistance, including PI3K/AKT/mTOR, JAK/STAT, Wnt/β-catenin, NOTCH, Hedgehog, and NF-κB [116]. Targeted inhibitors directed against these pathways are under investigation. For example, DCC-2036, a small-molecule tyrosine kinase inhibitor, blocks HCK and the PI3K/AKT/mTORC1 axis, thereby reducing OS cell proliferation, migration, invasion, and EMT while also inducing autophagy [117]. Similarly, inhibition of anti-apoptotic proteins such as MCL-1 using BH3 mimetics, in combination with regorafenib, enhances tumor cell death and improves survival in preclinical models of metastatic OS [118].

OS tumors are highly vascularized, making angiogenesis an important therapeutic target. VEGF inhibitors, including apatinib, can provide temporary disease stabilization; however, resistance frequently develops owing to tumor heterogeneity and adaptive signaling mechanisms [116,118]. TME plays a significant role in modulating disease progression and therapeutic response. Single-cell RNA sequencing studies have revealed substantial immune heterogeneity within OS tumors, with TIGIT^+^ T cells identified as potential targets for immunotherapy [119].

Autophagy recycles damaged cell components and plays a context-dependent role in OS. While it may suppress tumor initiation, established OS tumors often exploit autophagy to survive metabolic stress and chemotherapy [51,120]. Accordingly, pharmacological agents that either induce or inhibit autophagy are being evaluated as adjuncts to chemotherapy and targeted therapies to enhance OS treatment sensitivity.

### 6.3. Potential Future Therapies

Future therapeutic strategies [Table A1] for OS increasingly emphasize precision medicine, combination regimens, and targeting of the TME. Advanced molecular profiling, including genomic and transcriptomic analyses, enables treatment selection based on tumor-specific features and helps predict which patients are most likely to respond to targeted therapies [120].

Targeted approaches include tyrosine kinase inhibitors, PI3K/AKT/mTOR inhibitors, and immune checkpoint inhibitors, which are often combined to overcome tumor heterogeneity and therapeutic resistance [120]. Immunotherapeutic strategies, targeting inhibitory receptors, such as TIGIT, may restore T cell cytotoxicity, particularly when combined with other therapeutic modalities [120]. Modulation of autophagy remains a promising strategy [99,117]. Combining autophagy modulators with chemotherapy or targeted agents can increase tumor cell death and reduce the development of resistance. Targeting tumor metabolic dependencies, including BCL-2 family proteins, together with kinase inhibitors, can improve survival in models of metastatic OS [121]. Nanotechnology-based drug delivery systems, including liposomes and nanoparticles, can enhance tumor-specific drug accumulation while reducing systemic toxicity [120].

## 7. Conclusions

Therapeutic resistance stems from genetic changes inherent to the and from adaptive responses driven by the TME, highlighting the need for treatment strategies that extend beyond conventional chemotherapy to include targeted therapies, immunomodulatory approaches, and microenvironment-focused interventions. Advances in diagnostic-based therapy, along with precision medicine and innovative drug delivery systems, provide promising avenues to tailor treatments and overcome intratumoral heterogeneity.

The convergence of insights into cellular crosstalk, resistance mechanisms, and metastatic behavior underscores the importance of integrated, multipronged therapeutic strategies. Future research should continue to elucidate TME-driven mechanisms, identify actionable molecular targets, and translate these findings into clinically effective interventions. A deep understanding of tumor–microenvironment interactions will be essential for improving survival, reducing recurrence, and enhancing the quality of life of patients affected by this challenging malignancy.

## Data Availability

No new data were created or analyzed in this study.

## Abbreviations

The following abbreviations are used in this manuscript:

MDPI	Multidisciplinary Digital Publishing Institute
DOAJ	Directory of open access journals
TLA	Three-letter acronym
LD	Linear dichroism
TME	Tumor microenvironment
EV	Extracellular Vesicles
HUVECs	Exosomes derived from human umbilical vein ECs
CAFs	Cancer-Associated Fibroblasts
ECM	Extracellular Matrix
MATs	Mesenchymal–Amoeboid Transitions
MSCs	Mesenchymal Stromal Cells
BDNF	Brain-Derived Neurotrophic Factors
OSDCs	Osteosarcoma Derived Stromal Cells
CSC	Cancer Stem Cells
FD	Femoral Diaphysis
FE	Femoral Metaphysis
TANs	Tumor-Associated Neutrophils
NETs	Neutrophil Extracellular Traps
NLR	Neutrophil-to-Lymphocyte Ratio
TAMs	Tumor-Associated Macrophages
IGF1	Insulin-like Growth Factor
EMT	Epithelial–Mesenchymal Transition
Tregs	Regulatory CD4^+^ T cells
lncRNA	Long noncoding RNAs
PLEK	Pleckstrin
PLR	Platelet-to-Lymphocyte ratio
CAR	C-reactive protein-to-albumin ratio
ICD	Immunogenic Cell Death
MDSCs	Myeloid-Derived Suppressor Cells
EPCs	Endothelial Progenitor Cells
CTCs	Circulating Tumor Cells
ELA2	Neutrophil Elastase

## Appendix A

**Table A1:** This table summarizes potential future therapies, what they would target and what their mechanism would be.

Category	Target/Strategy	Mechanism/Notes
Precision Medicine	Genomic and transcriptomic profiling	Tailor therapy to tumor-specific features; predict response
Targeted Therapy	TKIs	Block growth signaling; dual VEGF/mTOR inhibition promising
Targeted Therapy	PI3K/AKT/mTOR inhibitors	Inhibit OS proliferation; often combined with other agents
Immunotherapy	Checkpoint inhibitors	Enhance anti-tumor immune activity
Immunotherapy	CAR-T cells	Redirect T cells to target OS cells
Immunotherapy	Macrophage reprogramming	Convert immunosuppressive macrophages to anti-tumor
Immunotherapy	TIGIT inhibition	Restore T cell cytotoxicity, effective in combinations
Nanotechnology	Liposomes/nanoparticles	Improve tumor-specific drug delivery; reduce toxicity
Autophagy Modulation	Autophagy enhancers/inhibitors	Alter OS survival under treatment stress; combine with chemo/targeted therapy
Metabolic Targeting	BCL-2 family + kinase inhibitors	Exploit tumor metabolic dependencies; improve survival in metastatic models
